# 2-Isopropyl-4-meth­oxy-5-methyl­phenyl acetate

**DOI:** 10.1107/S160053681302922X

**Published:** 2013-10-26

**Authors:** Radouane Oubabi, Aziz Auhmani, My Youssef Ait Itto, Mohamed Driss, El Hassane Soumhi

**Affiliations:** a Laboratoire de Synthése Organique et Physico-Chimie Moléculaire, Faculté des Sciences-Semlalia, Université Cadi Ayyad, BP 2390, 40001, Marrakech, Morocco; b Laboratoire de Matériaux et Cristallochimie, Faculté des Sciences de Tunis, Université de Tunis ElManar, 2092 ElManar II, Tunis, Tunisia; c Equipe de Chimie des Matériaux et de l’Environnement, FSTG-Marrakech, Université Cadi Ayyad, Bd Abdelkrim Khattabi, BP 549, Marrakech, Morocco

## Abstract

In the title compound, C_13_H_18_O_3_, the benzene ring is almost perpendicular to the acet­oxy plane, making a dihedral angle of 89.33 (11)°. In the crystal, mol­ecules are linked by weak C—H⋯O hydrogen bonds, forming a zigzag chain along the *c-*axis direction.

## Related literature

For background to natural monoterpenic phenols and their derivatives, see: Yuan-Lang & Erdtman (1962 ▶); Ündeğer *et al.* (2009 ▶); Osorio *et al.* (2006 ▶). For a related structure, see: Rajouani *et al.* (2008 ▶).
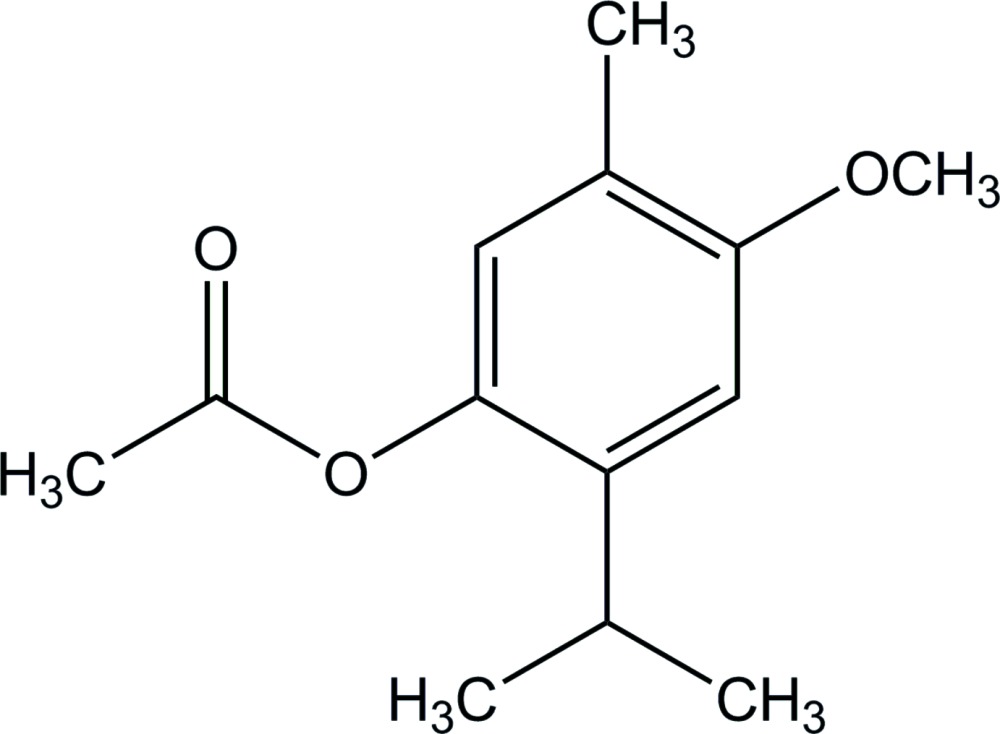


## Experimental

### 

#### Crystal data

C_13_H_18_O_3_
*M* *_r_* = 222.27
Monoclinic, 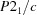

*a* = 10.829 (2) Å
*b* = 9.600 (2) Å
*c* = 12.530 (3) Å
β = 100.34 (2)°
*V* = 1281.4 (5) Å^3^
*Z* = 4
Mo *K*α radiation
μ = 0.08 mm^−1^
*T* = 300 K
0.3 × 0.15 × 0.1 mm

#### Data collection

Enraf–Nonius CAD-4 diffractometer
Absorption correction: ψ scan (North *et al.*, 1968 ▶) *T*_min_ = 0.521, *T*_max_ = 0.992
3366 measured reflections
2780 independent reflections
1594 reflections with *I* > 2σ(*I*)
*R*_int_ = 0.018
2 standard reflections every 60 min intensity decay: 1%

#### Refinement

*R*[*F* ^2^ > 2σ(*F* ^2^)] = 0.046
*wR*(*F* ^2^) = 0.132
*S* = 1.02
2780 reflections
151 parameters
H-atom parameters constrained
Δρ_max_ = 0.16 e Å^−3^
Δρ_min_ = −0.13 e Å^−3^

### 

Data collection: *CAD-4 EXPRESS* (Enraf–Nonius, 1989 ▶); cell refinement: *CAD-4 EXPRESS*; data reduction: *MolEN* (Fair, 1990 ▶); program(s) used to solve structure: *SHELXS97* (Sheldrick, 2008 ▶); program(s) used to refine structure: *SHELXL97* (Sheldrick, 2008 ▶); molecular graphics: *ORTEP-3 for Windows* (Farrugia, 2012 ▶); software used to prepare material for publication: *WinGX* (Farrugia, 2012 ▶).

## Supplementary Material

Crystal structure: contains datablock(s) I, hasacetf. DOI: 10.1107/S160053681302922X/is5314sup1.cif

Structure factors: contains datablock(s) I. DOI: 10.1107/S160053681302922X/is5314Isup2.hkl

Supplementary material file. DOI: 10.1107/S160053681302922X/is5314Isup3.cml

Additional supplementary materials:  crystallographic information; 3D view; checkCIF report

## Figures and Tables

**Table 1:** Hydrogen-bond geometry (Å, °)

*D*—H⋯*A*	*D*—H	H⋯*A*	*D*⋯*A*	*D*—H⋯*A*
C3—H1⋯O2^i^	0.93	2.60	3.523 (3)	171

Symmetry code: (i) 
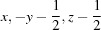
.

**Fig. 1.**
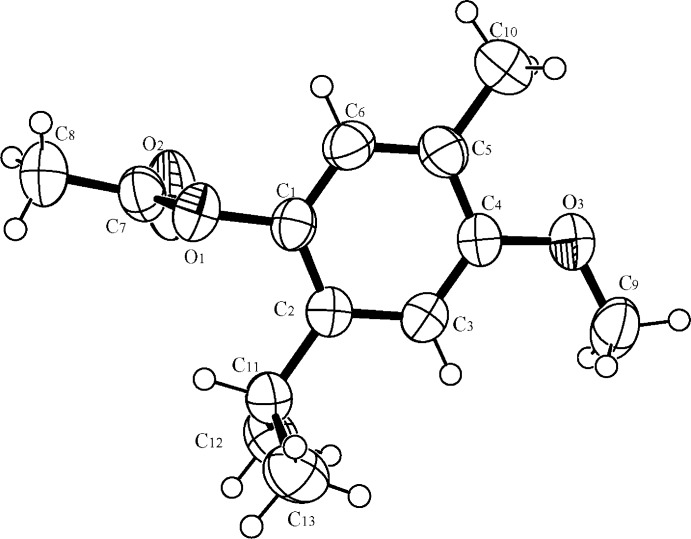
The molecule structure of the title compound with 50% probability ellipsoids.

## supplementary crystallographic information

### 1. Comment

The *p*-methoxythymol is a natural phenol extracted from heartwood of tetraclinis articulate (Yuan-Lang *et al.*, 1962). In several recent studies, natural monoterpenic phenols and their derivatives have revealed extensive biological activities, ranging from antimicrobial (Ündeğer *et al.*, 2009) to Antileishmanial and Cytotoxic activities (Osorio *et al.*, 2006). In the aim of preparing monoterpenic phenol derivatives, we report here, the hemisynthesis of 2-isopropyl-4-methoxy-5-methylphenyl acetate from naturally occurred *p*-methoxythymol. Thus, treatment of *p*-methoxythymol with acetic anhydride in pyridine, and provides the title compound as colorless crystals in 72.4% yield. Its structure was fully characterized by its mass and NMR spectroscopic data. Furthermore, the crystallographic study made it possible to determine the stereochemistry. The main geometric features of this group are in good agreement with those observed in a similar compound (Rajouani *et al.*, 2008).

### 2. Experimental

A solution of *p*-methoxythymol (111 mg, 0.617 mmol) in acetic anhydride (20 ml) and pyridine (20 ml) was heated under reflux for 24 h. After cooling, the mixture was acidified with 1 N HCl solution then extracted with ether (3 × 20 ml). The organic layer was washed with water, dried on anhydrous Na_2_SO_4_ and then evaporated under reduced pressure. The obtained residue was chromatographied on silica gel column using hexane and ethyl acetate (97/3) as eluent, to give 2-isopropyl-4-methoxy-5-methylphenyl acetate (100 mg) in 72.4% yield.

### 3. Refinement

All H-atoms were located in a difference map and refined using a riding model with C—H = 0.93–0.98 Å, and with *U*_iso_(H) = 1.2*U*_eq_(C).

### Crystal data

**Table:**

C_13_H_18_O_3_	*F*(000) = 480
*M**_r_* = 222.27	*D*_x_ = 1.152 Mg m^−^^3^
Monoclinic, *P*2_1_/*c*	Mo *K*α radiation, λ = 0.71073 Å
Hall symbol: -P 2ybc	Cell parameters from 25 reflections
*a* = 10.829 (2) Å	θ = 10–15°
*b* = 9.600 (2) Å	µ = 0.08 mm^−^^1^
*c* = 12.530 (3) Å	*T* = 300 K
β = 100.34 (2)°	Prism, colourless
*V* = 1281.4 (5) Å^3^	0.3 × 0.15 × 0.1 mm
*Z* = 4	

### Data collection

**Table:**

Enraf–Nonius CAD-4 diffractometer	1594 reflections with *I* > 2σ(*I*)
Radiation source: fine-focus sealed tube	*R*_int_ = 0.018
Graphite monochromator	θ_max_ = 27.0°, θ_min_ = 2.7°
ω/2θ scans	*h* = −13→1
Absorption correction: ψ scan (North *et al.*, 1968)	*k* = −1→12
*T*_min_ = 0.521, *T*_max_ = 0.992	*l* = −15→15
3366 measured reflections	2 standard reflections every 60 min
2780 independent reflections	intensity decay: 1%

### Refinement

**Table:**

Refinement on *F*^2^	Secondary atom site location: difference Fourier map
Least-squares matrix: full	Hydrogen site location: difference Fourier map
*R*[*F*^2^ > 2σ(*F*^2^)] = 0.046	H-atom parameters constrained
*wR*(*F*^2^) = 0.132	*w* = 1/[σ^2^(*F*_o_^2^) + (0.0525*P*)^2^ + 0.2201*P*] where *P* = (*F*_o_^2^ + 2*F*_c_^2^)/3
*S* = 1.02	(Δ/σ)_max_ < 0.001
2780 reflections	Δρ_max_ = 0.16 e Å^−^^3^
151 parameters	Δρ_min_ = −0.13 e Å^−^^3^
0 restraints	Extinction correction: *SHELXL97* (Sheldrick, 2008), Fc^*^=kFc[1+0.001xFc^2^λ^3^/sin(2θ)]^-1/4^
Primary atom site location: structure-invariant direct methods	Extinction coefficient: 0.016 (3)

### Special details

**Table:**

Geometry. All e.s.d.'s (except the e.s.d. in the dihedral angle between two l.s. planes) are estimated using the full covariance matrix. The cell e.s.d.'s are taken into account individually in the estimation of e.s.d.'s in distances, angles and torsion angles; correlations between e.s.d.'s in cell parameters are only used when they are defined by crystal symmetry. An approximate (isotropic) treatment of cell e.s.d.'s is used for estimating e.s.d.'s involving l.s. planes.
Refinement. Refinement of *F*^2^ against ALL reflections. The weighted *R*-factor *wR* and goodness of fit *S* are based on *F*^2^, conventional *R*-factors *R* are based on *F*, with *F* set to zero for negative *F*^2^. The threshold expression of *F*^2^ > σ(*F*^2^) is used only for calculating *R*-factors(gt) *etc*. and is not relevant to the choice of reflections for refinement. *R*-factors based on *F*^2^ are statistically about twice as large as those based on *F*, and *R*- factors based on ALL data will be even larger.

### Fractional atomic coordinates and isotropic or equivalent isotropic displacement parameters (Å^2^)

**Table:**

	*x*	*y*	*z*	*U*_iso_*/*U*_eq_	
O1	0.76460 (12)	0.08305 (13)	0.33870 (9)	0.0587 (4)	
O2	0.76646 (18)	−0.07552 (19)	0.46746 (13)	0.1062 (7)	
O3	0.43208 (12)	−0.20942 (15)	0.03401 (10)	0.0673 (4)	
C1	0.68418 (16)	−0.00154 (18)	0.26312 (13)	0.0496 (4)	
C2	0.73387 (16)	−0.09160 (17)	0.19660 (13)	0.0476 (4)	
C3	0.64806 (16)	−0.16266 (18)	0.11839 (14)	0.0522 (4)	
H1	0.6777	−0.2242	0.0715	0.063*	
C4	0.52031 (16)	−0.14316 (18)	0.10945 (13)	0.0514 (4)	
C5	0.47183 (17)	−0.0533 (2)	0.17877 (14)	0.0562 (5)	
C6	0.55666 (17)	0.0175 (2)	0.25510 (14)	0.0570 (5)	
H2	0.5272	0.0792	0.3020	0.068*	
C7	0.79534 (17)	0.0364 (2)	0.44091 (15)	0.0612 (5)	
C8	0.8695 (2)	0.1412 (3)	0.51249 (17)	0.0803 (7)	
H3	0.8841	0.1081	0.5860	0.096*	
H4	0.8239	0.2274	0.5082	0.096*	
H5	0.9485	0.1559	0.4896	0.096*	
C9	0.4748 (2)	−0.2910 (2)	−0.04666 (16)	0.0742 (6)	
H6	0.5283	−0.2355	−0.0830	0.089*	
H7	0.4041	−0.3229	−0.0983	0.089*	
H8	0.5210	−0.3697	−0.0132	0.089*	
C10	0.33271 (18)	−0.0334 (3)	0.16960 (19)	0.0812 (7)	
H9	0.3168	0.0323	0.2233	0.097*	
H10	0.2943	−0.1210	0.1810	0.097*	
H11	0.2982	0.0011	0.0986	0.097*	
C11	0.87402 (16)	−0.11360 (19)	0.20594 (15)	0.0546 (5)	
H12	0.9162	−0.0529	0.2641	0.066*	
C12	0.9114 (2)	−0.2629 (2)	0.23722 (18)	0.0747 (6)	
H13	0.8718	−0.3250	0.1813	0.090*	
H14	0.8850	−0.2857	0.3043	0.090*	
H15	1.0009	−0.2723	0.2458	0.090*	
C13	0.9195 (2)	−0.0725 (3)	0.10231 (18)	0.0785 (6)	
H16	1.0093	−0.0797	0.1133	0.094*	
H17	0.8949	0.0218	0.0837	0.094*	
H19	0.8830	−0.1335	0.0445	0.094*	

### Atomic displacement parameters (Å^2^)

**Table:**

	*U*^11^	*U*^22^	*U*^33^	*U*^12^	*U*^13^	*U*^23^
O1	0.0685 (8)	0.0536 (7)	0.0512 (7)	−0.0086 (6)	0.0036 (6)	−0.0056 (6)
O2	0.1266 (15)	0.1007 (13)	0.0758 (10)	−0.0443 (11)	−0.0236 (10)	0.0304 (10)
O3	0.0564 (7)	0.0792 (9)	0.0621 (8)	−0.0073 (7)	−0.0007 (6)	−0.0123 (7)
C1	0.0553 (10)	0.0460 (10)	0.0448 (9)	−0.0017 (8)	0.0022 (8)	0.0014 (8)
C2	0.0509 (9)	0.0444 (9)	0.0464 (9)	−0.0011 (8)	0.0054 (7)	0.0033 (8)
C3	0.0574 (11)	0.0510 (10)	0.0475 (9)	−0.0004 (8)	0.0079 (8)	−0.0034 (8)
C4	0.0508 (10)	0.0521 (10)	0.0481 (9)	−0.0037 (8)	0.0006 (8)	0.0019 (8)
C5	0.0516 (10)	0.0583 (11)	0.0562 (10)	0.0076 (9)	0.0030 (8)	0.0052 (9)
C6	0.0624 (11)	0.0543 (11)	0.0543 (11)	0.0077 (9)	0.0108 (9)	−0.0040 (9)
C7	0.0533 (11)	0.0727 (13)	0.0545 (11)	−0.0043 (10)	0.0013 (9)	0.0009 (10)
C8	0.0706 (13)	0.0957 (17)	0.0675 (13)	−0.0095 (12)	−0.0071 (11)	−0.0155 (12)
C9	0.0796 (14)	0.0819 (15)	0.0586 (11)	−0.0200 (12)	0.0054 (10)	−0.0148 (11)
C10	0.0567 (12)	0.0940 (17)	0.0900 (16)	0.0120 (12)	0.0052 (11)	−0.0100 (13)
C11	0.0504 (10)	0.0560 (11)	0.0559 (10)	−0.0048 (9)	0.0053 (8)	−0.0048 (9)
C12	0.0615 (12)	0.0719 (14)	0.0860 (15)	0.0071 (11)	0.0006 (11)	0.0076 (12)
C13	0.0652 (13)	0.0903 (16)	0.0839 (15)	−0.0030 (12)	0.0236 (11)	0.0106 (13)

### Geometric parameters (Å, º)

**Table:**

O1—C7	1.341 (2)	C11—C12	1.522 (3)
O1—C1	1.421 (2)	C8—H3	0.9600
O2—C7	1.183 (2)	C8—H4	0.9600
O3—C4	1.374 (2)	C8—H5	0.9600
O3—C9	1.420 (2)	C9—H6	0.9600
C1—C2	1.376 (2)	C9—H7	0.9600
C1—C6	1.379 (2)	C9—H8	0.9600
C2—C3	1.401 (2)	C10—H9	0.9600
C2—C11	1.516 (2)	C10—H10	0.9600
C3—C4	1.380 (2)	C10—H11	0.9600
C3—H1	0.9300	C11—H12	0.9800
C4—C5	1.392 (3)	C12—H13	0.9600
C5—C6	1.380 (2)	C12—H14	0.9600
C5—C10	1.502 (3)	C12—H15	0.9600
C6—H2	0.9300	C13—H16	0.9600
C7—C8	1.484 (3)	C13—H17	0.9600
C11—C13	1.521 (3)	C13—H19	0.9600
			
C7—O1—C1	117.57 (14)	O3—C9—H7	109.5
C4—O3—C9	118.02 (15)	H6—C9—H7	109.5
C2—C1—C6	122.33 (16)	O3—C9—H8	109.5
C2—C1—O1	120.23 (15)	H6—C9—H8	109.5
C6—C1—O1	117.31 (16)	H7—C9—H8	109.5
C1—C2—C3	116.58 (16)	C5—C10—H9	109.5
C1—C2—C11	122.40 (15)	C5—C10—H10	109.5
C3—C2—C11	121.02 (16)	H9—C10—H10	109.5
C4—C3—C2	121.33 (17)	C5—C10—H11	109.5
C4—C3—H1	119.3	H9—C10—H11	109.5
C2—C3—H1	119.3	H10—C10—H11	109.5
O3—C4—C3	123.79 (16)	C2—C11—C13	111.77 (15)
O3—C4—C5	115.01 (16)	C2—C11—C12	111.54 (15)
C3—C4—C5	121.20 (16)	C13—C11—C12	110.65 (17)
C6—C5—C4	117.32 (16)	C2—C11—H12	107.5
C6—C5—C10	121.61 (18)	C13—C11—H12	107.5
C4—C5—C10	121.07 (17)	C12—C11—H12	107.5
C1—C6—C5	121.23 (17)	C11—C12—H13	109.5
C1—C6—H2	119.4	C11—C12—H14	109.5
C5—C6—H2	119.4	H13—C12—H14	109.5
O2—C7—O1	122.63 (18)	C11—C12—H15	109.5
O2—C7—C8	125.96 (19)	H13—C12—H15	109.5
O1—C7—C8	111.41 (18)	H14—C12—H15	109.5
C7—C8—H3	109.5	C11—C13—H16	109.5
C7—C8—H4	109.5	C11—C13—H17	109.5
H3—C8—H4	109.5	H16—C13—H17	109.5
C7—C8—H5	109.5	C11—C13—H19	109.5
H3—C8—H5	109.5	H16—C13—H19	109.5
H4—C8—H5	109.5	H17—C13—H19	109.5
O3—C9—H6	109.5		
			
C7—O1—C1—C2	−95.9 (2)	C3—C4—C5—C6	1.3 (3)
C7—O1—C1—C6	88.2 (2)	O3—C4—C5—C10	0.3 (3)
C6—C1—C2—C3	0.8 (3)	C3—C4—C5—C10	−179.34 (18)
O1—C1—C2—C3	−174.89 (14)	C2—C1—C6—C5	−0.3 (3)
C6—C1—C2—C11	−179.47 (17)	O1—C1—C6—C5	175.57 (16)
O1—C1—C2—C11	4.8 (2)	C4—C5—C6—C1	−0.8 (3)
C1—C2—C3—C4	−0.3 (2)	C10—C5—C6—C1	179.85 (19)
C11—C2—C3—C4	179.98 (16)	C1—O1—C7—O2	6.1 (3)
C9—O3—C4—C3	−7.0 (3)	C1—O1—C7—C8	−174.65 (16)
C9—O3—C4—C5	173.38 (16)	C1—C2—C11—C13	−118.73 (19)
C2—C3—C4—O3	179.64 (15)	C3—C2—C11—C13	61.0 (2)
C2—C3—C4—C5	−0.8 (3)	C1—C2—C11—C12	116.80 (19)
O3—C4—C5—C6	−179.06 (15)	C3—C2—C11—C12	−63.5 (2)

### Hydrogen-bond geometry (Å, º)

**Table:**

*D*—H···*A*	*D*—H	H···*A*	*D*···*A*	*D*—H···*A*
C3—H1···O2^i^	0.93	2.60	3.523 (3)	171

Symmetry code: (i) *x*, −*y*−1/2, *z*−1/2.

## References

[bb1] Enraf–Nonius (1989). *CAD-4 EXPRESS* Enraf–Nonius, Deft, The Netherlands.

[bb2] Fair, C. K. (1990). *MolEN* Enraf–Nonius, Delft, The Netherlands.

[bb3] Farrugia, L. J. (2012). *J. Appl. Cryst.* **45**, 849–854.

[bb4] North, A. C. T., Phillips, D. C. & Mathews, F. S. (1968). *Acta Cryst.* A**24**, 351–359.

[bb5] Osorio, E., Arango, G., Robledo, S., Muñoz, D., Jaramillo, L. & Vélez, I. (2006). *Acta Farm. Bonaer.* **25**, 405–413.

[bb6] Rajouani, N., Ait Itto, My. Y., Benharref, A., Auhmani, A. & Daran, J.-C. (2008). *Acta Cryst.* E**64**, o762. 10.1107/S1600536808007769 PMC2960938 21202151

[bb7] Sheldrick, G. M. (2008). *Acta Cryst.* A**64**, 112–122. 10.1107/S0108767307043930 18156677

[bb8] Ündeğer, Ü., Başaran, A., Degen, G. H. & Başaran, N. (2009). *Food Chem. Toxicol.* **47**, 2037–2043. 10.1016/j.fct.2009.05.020 19477215

[bb9] Yuan-Lang, C. & Erdtman, H. (1962). *Acta Chem. Scand.* **16**, 1291–1295.

